# Abstract of the Proceedings of the Buffalo Medical Association, Reported by the Secretary

**Published:** 1859-02

**Authors:** Austin Flint


ART. VI.—*Abstract of Proceedings of the Buffalo Medical Association.*

TUESDAY EVENING, Jan. 4, 1859.

The Association met.

Present—The President, Dr. Wyckoff in the chair; Drs. Lemon, Rochester, Miner, Wilcox, Whitney, White, Butler, Nott, and Flint, Jr.

The minutes of the last meeting were read and approved.

Prof. WHITE exhibited an interesting specimen of monstrosity. It was the offspring of a female aged 36 years. She menstruated last May, and became pregnant in June. In August and September she had some flowing. A specular examination was then made, and ulcerations were found on the os uteri, which presented a bleeding surface. Upon the application of the solid nitrate of silver, the hæmorrhage was arrested. The patient gained strength and was well up to the middle of December, when she was suddenly attacked with labor pains, and threw off the product which was exhibited to the Association.

The specimen was very curious; there was only half of the cranial surface, the remainder being entirely wanting; the legs were those of a six months fœtus, while the body was not larger than that of one at three months. There was the rudiment of one ear, and a single perfect eye, which was situated on the top of the head. The cord was wound tightly around the neck, nearly dividing it. The placenta had undergone extensive fatty degeneration. The woman had before been delivered of seven or eight healthy and well-formed children.

Prof. White was of the opinion that the woman conceived in May, and that the development of the lower extremities had not been interfered with; which accounted for their large size in proportion to the other organs. It was impossible to say how much the monstrosity was due to a diseased condition of the uterus; but much was undoubtedly attributable to the fatty condition of the placenta. Most monstrosities are due to the enclosure of two ova in the same sac; but Dr. White was at a loss to account for the absence of half the head and the remarkable misplacement of the eye. In regard to the treatment of the uterine difficulty during the gestation, he was of the opinion that labor was arrested, rather than excited, by the application of the caustic. This had been the result of his experience in many cases; and he had, indeed, prevented abortion by that means.

Prof. ROCHESTER remarked, that he called to mind two cases of hæmorrhage during the early months of pregnancy, partial deformity of the children following; this may have been due to the flowing.

In the first case, the application of the nitrate of silver to the congested os uteri, relieved the flooding; the infant had a curvature of one of the limbs. The second case was a confirmation of the proposition of Dr. White.

Dr. MINER inquired if Dr. White, in his theory of the common formation of monstrosities, ignored the common impression that many of them were due to mental emotions of the mother: for example, a woman would insist that her child would have a hare lip, because, during gestation, she had seen a person with such deformity; and the result was as she had predicted. He also inquired if cauterization of the neck of the uterus, during gestation, was never attended with danger.

Prof. WHITE replied that he was correctly understood when he said that most monstrosities were due to a fusion of two ova. In regard to the effect of mental emotion of the mother in producing malformations of the fœtus, he could not say positively that it was so, but the supposition could not be entirely put aside. He had witnessed many cases where women had correctly predicted maiformations, and many others in which these malformations did not occur in accordance with such predictions. In regard to the application of the nitrate of silver to the os and cervix uteri of a pregnant female, he always exercised a discretion; but he thought that in cases of ulceration during the early period of gestation, such treatment was generally beneficial. He had an illustration of this within the last few weeks. In the case of a woman who was in her fifth pregnancy and had always before suffered from excessive nausea, which was especially troublesome at this time, he had applied the solid nitrate to the os, although she had passed the fifth month, relieving the nausea, and producing no unpleasant consequences. He never has any fear of applying this agent moderately to the outside of the neck, never passing the stick within the cavity, and it usually acts merely as a sedative. He is not in the habit of using the purest preparation of silver, but an agent which contains forty-nine per cent. of the nitrate of potassa, which renders it harder and much less active.

Prof. ROCHESTER gave an account of an obstetrical case which he had seen in consultation with Dr. GOULD. The patient was delivered by Mr. Robinson, at the request of Dr. Gould, who was unable, from sickness, to attend her, and an ordinary bandage was applied. The next day she was very ill; had tympanitis, high pulse, etc. On removing the bandage, Mr. Robinson discovered a tumor which he supposed to be the enlarged uterus, but he was unable to entirely satisfy himself of its character. Dr. Rochester then saw the patient with Dr. Gould. The tumor was nearly the size of a man's head, circumscribed and pyriform; its culminating point being the umbilicus. The probability of an umbilical hernia occurred to Dr. Rochester, which proved to be the case, as it was easily reduced. Afterwards there was no difficulty in determining the nature of the case. The woman died on the next day of metro-peritonitis.

Prof. ROCHESTER made a few remarks on the subject of vaccination. He thought that the mode which was generally employod in this country was by no means the best. He regarded the effects of vaccination with scab and lymph as quite different, especially in the protective power. He had lately re-vaccinated about one hundred persons, and had been successful in a much larger proportion of cases than he had expected. This he attributed to the imperfect result of the first vaccination with the crust, which is the ordinary mode. He also thought that the prevalence of variola resulted from this same cause. The vaccination with the crust might be good, but the protection was not perfect in some cases. If the lymph be taken at the proper time, there will be no admixture of pus, and the course of the vaccination, with such matter, will generally be more prompt and rapid, usually gaining one day. Where the crust is used, we frequently have an irritation as from a foreign body, which will interfere with the process of absorbtion. His success in re-vaccinating had been about one in six, when it had been done with lymph obtained at the fifth or sixth day, before there was a well marked areola. He obtained the matter from a slight puncture, by collecting it on quills which had been slightly roughened by scraping. With pure lymph he had never failed; but had been repeatedly unsuccessful with the crust. Dr. Rochester claimed that the protection from a vaccination with lymph lasted through life.

Dr. Miner said that the objection to the practice of vaccinating with lymph was the difficulty of keeping it fresh; in the summer, it was impossible to preserve it for more than three weeks. He preferred to collect it on the seventh or eighth day.

Dr. White remarked that his experience had commenced in 1832, with vaccinating with lymph which he had collected on threads and quills; and he had sometimes vaccinated from a fresh arm; he found it, however, very troublesome to obtain and preserve the virus, and soon began to use the crust. He now used the powdered crust with satisfactory results; this he introduced under the skin with a small silver canula. He thought it a very good idea to make the crust into a paste with glycerine; this had been mentioned to him by Prof. Moore, and he thought it worthy of a fair trial.

Dr. Mead was proposed for membership.

The Association then adjourned.

AUSTIN FLINT, Jr., M. D.,
Secretary.

